# Versatile Dicyanomethylene-Based Fluorescent Probes for the Detection of β-Amyloid in Alzheimer’s Disease: A Theoretical Perspective

**DOI:** 10.3390/ijms23158619

**Published:** 2022-08-03

**Authors:** Mengna Zhang, Haoqing Fu, Wei Hu, Jiancai Leng, Yujin Zhang

**Affiliations:** International School for Optoelectronic Engineering, School of Electrical Engineering and Automation, School of Chemistry and Chemical Engineering, Qilu University of Technology (Shandong Academy of Sciences), Jinan 250353, China; zzmn_131@163.com (M.Z.); fhq202243@163.com (H.F.); weihu@qlu.edu.cn (W.H.)

**Keywords:** fluorescent probe, β-amyloid, donor effect

## Abstract

Motivated by the growing demand for target chemosensors designed with diagnostic or therapeutic capability for fibrils related to amyloidosis diseases, we investigated in the present work the response mechanism of dicyanomethylene-based fluorescent probes for amyloid fibril using a combined approach, including molecular docking, quantum mechanics/molecular mechanics (QM/MM), and the quantum chemical method. Various binding modes for the probes in β-amyloid (Aβ) are discussed, and the fibril environment-induced molecular optical changes at the most stable site are compared to the fibril-free situation in aqueous environments. The results reveal that the fluorescence enhancement for the probes in Aβ observed experimentally is an average consequence over multiple binding sites. In particular, the conformational difference, including conjugation length and donor effect, significantly contributes to the optical property of the studied probes both in water and fibril. To further estimate the transition nature of the molecular photoabsorption and photoemission processes, the hole-electron distribution and the structural variation on the first excited state of the probes are investigated in detail. On the basis of the calculations, structure–property relationships for the studied chemosensors are established. Our computational approach with the ability to elucidate the available experimental results can be used for designing novel molecular probes with applications to Aβ imaging and the early diagnosis of Alzheimer’s disease.

## 1. Introduction

Dementia is now the seventh leading cause of mortality globally, and it has one of the highest costs to society [1]. Alzheimer’s disease (AD), as one of the neurodegenerative diseases that cannot be prevented, cured or even slowed down, has become a common major disease that threatens human life and health. The distinguishing feature of Alzheimer’s disease is the presence of β-amyloid (Aβ) that build up in the brain to the point that they obstruct normal cognitive functions. This usually manifests with changes in memory, abstract thinking, judgement, behavior, and emotions, and it ultimately interferes with physical control over the living body [2,3,4,5,6]. Thus, it is of great significance to achieve accurate monitoring of Aβ in the early stage of AD for prevention and treatment.

A variety of detection methods have been reported for the analysis of active substance amyloid related to AD [7,8,9,10]. Among them, fluorescence microscopy with advantages, such as a simple operation and a high temporal and spatial resolution, is widely used in biomedicine, biodetection, and other fields [11,12]. In this context, a number of fluorescent probes based on organic small molecules for sensing Aβ are developed in consideration of their low cost and facile synthesis [13,14,15]. For example, a series of push–pull compounds containing thioflavin T (ThT), with high selectivity were reported for fluorescence imaging Aβ plaques in vivo. Similarly, curcumin-based near-infrared Aβ fluorescence probes were designed by introducing terminal electron donor and acceptor groups, which showed significant fluorescence enhancement with the presence of Aβ fibrils. In particular, dicyanomethylene has been widely used as the electron acceptor group to construct donor–acceptor architectures with the ability to selectively bind to Aβ aggregates companied with strong fluorescence enhancement [16,17]. Cui and coworkers reported a series of novel near-infrared (NIR) Aβ probes named as DANIR 2a-c which were constructed by the *N*,*N*-dimethylaminophenyl-donating group and dicyanomethylene-accepting group bridged by π-conjugated double bonds. It showed that the probe with the longest polyenic chain exhibited high affinity and maximum emission in Aβ plaques [18]. In order to improve the intramolecular charge transfer and detection efficiency of these probes, DANIR 3a-e were synthesized by replacing the benzene ring with a naphthalene ring in DANIR 2a-c [19]. Moreover, DANIR 8-9 were designed by adopting the hydroxyethyl group instead of a methyl substituent in the donor segment [9]. Recently, many excellent results on dicyanomethylene-based Aβ fluorescent probes have been reported. Desirable affinity and lipophilicity were achieved for a dicyanomethylene-4H-pyran-based push–pull fluorophore, named PAD-1, which could specifically label and image cerebral Aβ fibrils in mouse models [20]. In addition, Ono et al. used a dimethylaminothienyl moiety as the electron-donating group to synthesize a new series of polyenic derivatives named as DTM-ns for the detection of Aβ [21]. Kim designed highly π-extended dipolar acedan fluorophores to shift the two-photon excited emission into the near-infrared regime to avoid autofluorescence for imaging Aβ plaques in tissue [22].

Although the widespread experimental measurements emphasize the high affinities and excellent fluorescent properties for the dicyanomethylene-containing Aβ probes, it is necessary to have an in-depth understanding of the nature of interactions between the biostructure and sensors, further revealing the inner mechanisms. More importantly, there is increasing demand for comparing the properties of the probes at the same level. Based on this knowledge, one can propose the structure–property relationship and further provide design strategies for Aβ fluorescent probe with highly sensitive and rapid fluorescence response. Motivated by these scientific issues, theoretical investigations are highly desired. However, the research on this aspect is deficient on account of the computational limitation of simulating such a multiscale fibril–ligand complex. In this work, an integrated approach, including molecular docking, quantum mechanics/molecular mechanics (QM/MM) method, and quantum chemical calculation, is employed to study the interaction of the fibril–probe complexes and amyloid-specific optical properties of versatile dicyanomethylene-based fluorescent probes [9,19,23,24,25]. Binding modes of the probes with Aβ fibril are analyzed and the fibril–probe interactions are evaluated by binding energies. According to the statistics, the most likely binding sites for the chemosensors are obtained. Furthermore, optical properties for the probes both in aqueous phase and in Aβ are studied by adopting the quantum chemical calculation and QM/MM method, respectively. Excited state dynamics investigations are carried out to illustrate the fluorescent characteristics of the studied objects. Thus, donor effect and the influence of spacer length on the photophysical properties are highlighted, which can provide valuable information on design principles for β-amyloid chemosensors with high affinity and sensitivity.

## 2. Results and Discussion

### 2.1. Conformation and Binding Site Analysis

**Scheme 1:**
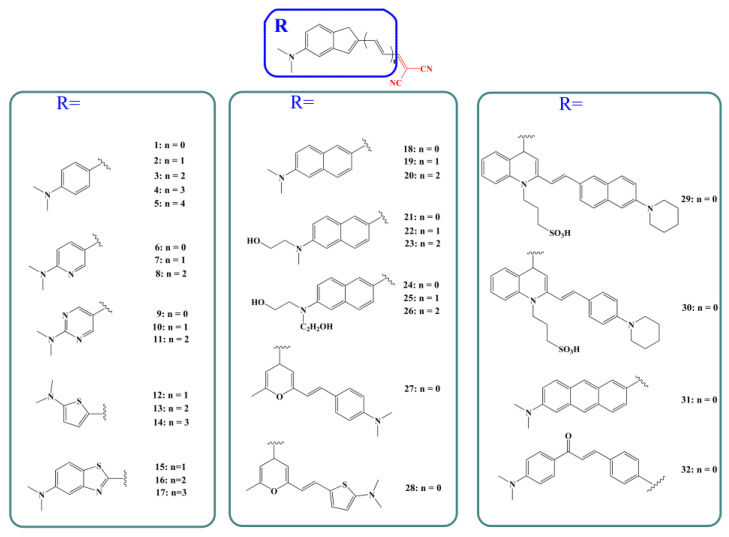
Schematic structure of molecular probes (labeled as probes 1–32 hereafter) studied in the present work.

The primary goal of this work is to study the interaction of dicyanomethylene-based fluorescent probes with Aβ fibril and to investigate the β-amyloid specific optical properties of the probes to elucidate the responsive mechanism. Fourteen substituent groups were chosen as the electron donor to connect with the dicyanomethylene moiety through the polyene chain. By varying the length for the polyene spacer, a total of 32 probe models which had been synthesized and characterized experimentally [9,19,23,24,25] were adopted (labeled as probes 1–32, shown in Scheme 1).

**Figure 1:**
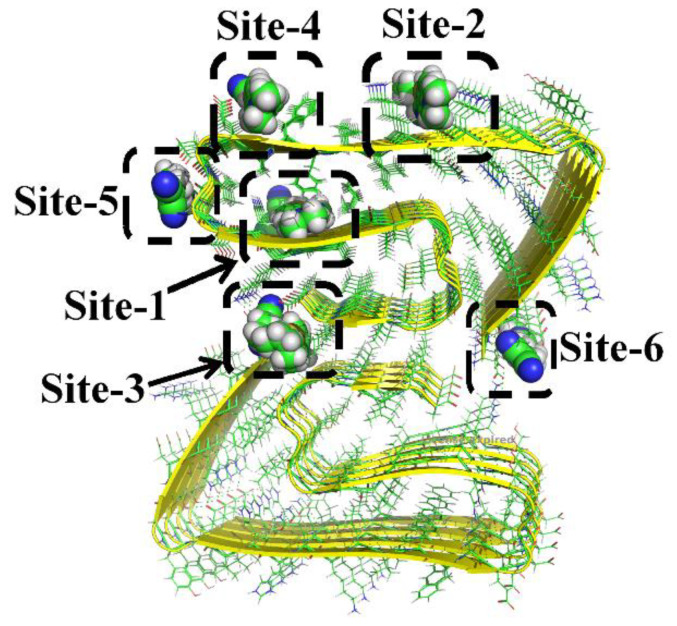
Various binding sites of the probes in β-amyloid (taken probe 1 as the example).

**Figure 2:**
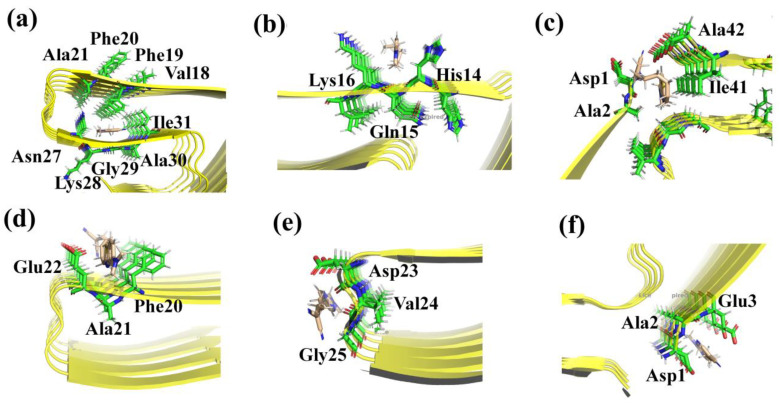
Detailed binding sites for a probe in β-amyloid (**a**) Site-1, (**b**) Site-2, (**c**) Site-3, (**d**) Site-4, (**e**) Site-5, and (**f**) Site-6. The fibril is shown in cartoon mode, and some important residues are shown in stick mode.

Docking simulations show that the studied chemosensors can be mainly docked onto six different sites in the fibril (shown in Figure 1). Thereinto, only one binding site is completely a core site (Site-1), four sites appear on the surface (Site-2, Site-4, Site-5, and Site-6), and Site-3 is in the interfacial region. The detailed insets of multiple binding sites in Figure 2a show that Site-1, composed of the residues Val18, Phe19, Phe20, Lys28, Gly29 and Ala30, is deeply buried inside the fibril with a highly hydrophobic environment. Site-2 and Site-4 are formed by side chains of His14, Gln15, Lys16 and Phe20, Ala21, and Glu22, respectively. Additionally, the backbone atoms of Asp23, Val24, and Gly25 formed the other site on the surface (Site-5). Unlike the above-mentioned surface sites, Site-6 consists of terminal residues (Asp1, Ala2, and Glu3). Similarly, Site-3 is also made up of terminal residues, including Asp1, Ala2, Ile41, and Ala42. As there are no aromatic residues in Site-3, Site-5, and Site-6, π–π stacking interaction between the probe and the amyloid is negligible in these sites.

**Figure 3:**
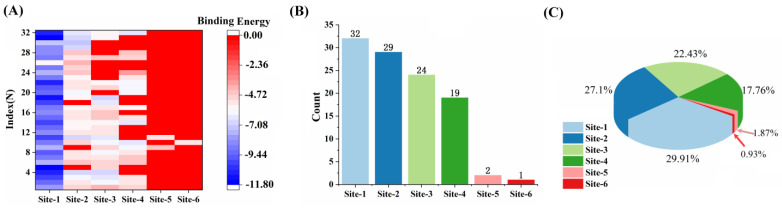
(**A**) Binding energy for probes 1–32 on different binding sites in Aβ. (**B**) The count numbers among all possible binding sites for the probes in Aβ. (**C**) The proportion of multiple binding sites among all possible binding sites.

Clustering of multiple binding sites with binding energy for each binding mode is collected in Figure 3A. It shows that the binding free energies of the probes with Aβ fibril in Site-1 are much lower than that in other sites, which is due to the fact that they interact with more residues in the core site than the surface or the interfacial sites. Computed from the molecular docking results, the binding energies for probe 1 in Site-1 to Site-4 are −7.88, −4.87, −4.04, −5.11 kcal/mol, respectively, suggesting various interactions between the ligand and the fibril in different binding sites. The corresponding inhibition constants are 1.67, 268.95, 1100, and 180.81 μM in the simulation, while the experimental dissociation constant (K_d_) has been reported to be 1.95 μM, indicating that Site-1 is probably responsible for the probe binding in nature. Thus, one can judge that Site-1 is the dominant binding site, while other binding sites play incidental roles. For each series of the investigated probe in the same site, the binding energy is much lower for the probe with a longer polyene chain than that with a shorter chain. For example, binding energies for probes 1 to 5 in Site-1 are −7.88, −9.08, −9.88, −11.01, and −11.73 kcal/mol, respectively; and, for probes 6–8, the values are −7.53, −8.66, and −9.46 kcal/mol, respectively. This is in good agreement with experimental observations that probes with longer conjugated π system display a higher affinity to Aβ [26]. Therefore, the electron donor and the spacer length have a great impact on the interaction and the binding mode of the complexes.

By counting the binding sites for all probes in Aβ (see Figure 3B,C), the most favorable configuration for each probe is determined. Site-1 is the site associated with larger binding affinity, which may be dominant by the cavity volume and the composed residues of this site. Compared with the other binding sites, Site-1 is the only site available for every probe with a maximum ratio of approximately 30% among all sites. Next, we will focus on the Aβ-specific optical properties for the probes in Site-1.

### 2.2. β-Amyloid Induced Changes in Absorption

When studying the photophysics processes, including absorption and emission, oscillator strength defined as $\delta =\frac{2\omega_{f}}{3}{\sum_{\alpha }\left|\langle i|\mu_{\alpha }|f\rangle \right|}^{2}$, is utilized to describe the transition probability. Here, *ω_f_* and *μ_α_* denote the excited energy of the state *f* and the electric dipole moment operator in the direction *α*, respectively. To elucidate the character of the photoabsorption of the probes both in aqueous and in Aβ, we carried out optical property calculations using two different approaches, namely, the quantum chemical method and the QM/MM framework, as mentioned above.

**Figure 4:**
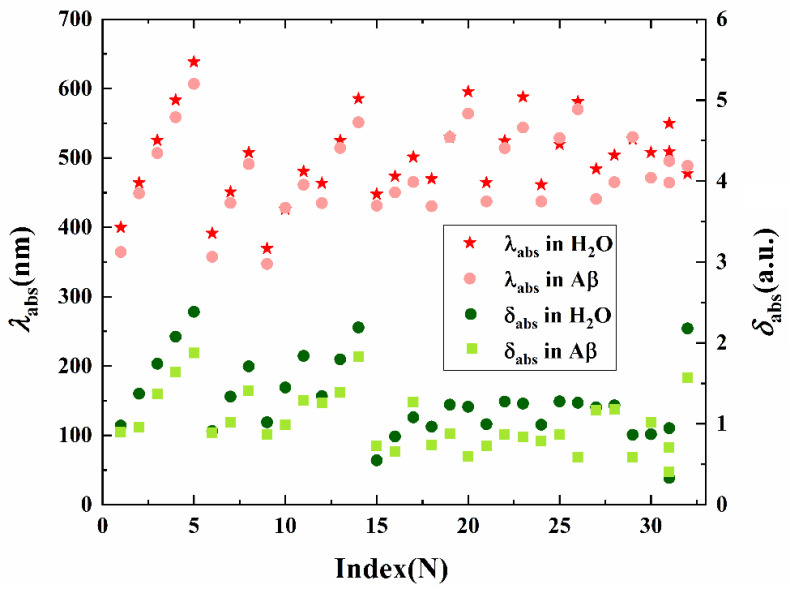
The absorption wavelength *λ*_abs_ (the left vertical axis) and oscillator strength *δ*_abs_ (the right vertical axis) for probes 1–32 in H_2_O and in Aβ.

In all cases except for probe 31, the absorption spectra in both aqueous and fibril exhibit one intense band in the UV or visible region, while probe 31 possesses two absorption bands locating at 550 and 509 nm in H_2_O, and 495 and 464 nm in Aβ, respectively, as plotted in Figure 4. Interestingly, the position of the absorption peak shows a red shift upon lengthening of the conjugate, both in H_2_O and in Aβ. However, the variation tendency of the absorption intensity is inconsistent with the increase in the spacer length.

**Figure 5:**
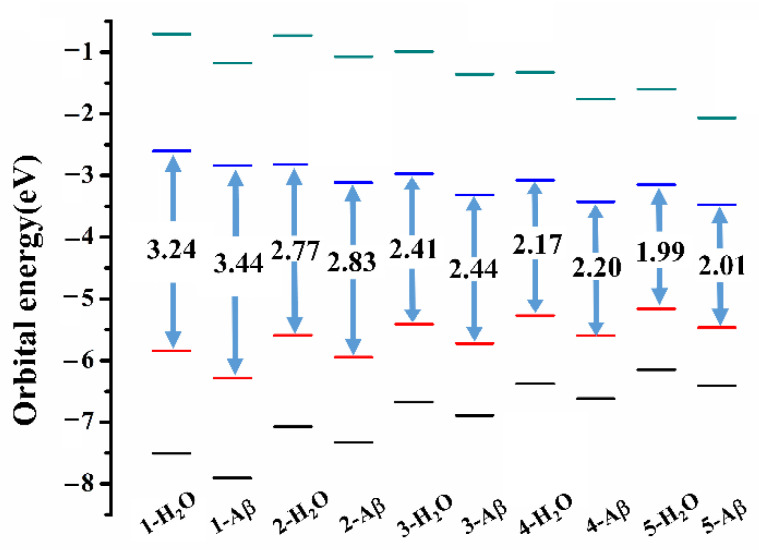
The energy levels of the molecular frontier orbitals and energy gap for probes 1–5 in different microenvironments.

The calculated wavelength corresponding to the vertical electronic transition for selected probes is compared to the experimental values on the absorption maxima in Table 1. Compounds 1 to 5 with different polyene chains exhibit maximum absorption wavelength from 400 nm to 638 nm with enhanced absorption intensity in H_2_O. Upon binding with Aβ, the absorption maxima are blue shifted and the intensity is reduced in comparison with those in aqueous on account of the interaction between the molecule and the fibril. Figure 5 displays the energy levels of the molecular frontier orbitals in different microenvironments for probes 1–5. The energy gap between the highest occupied molecular orbital (HOMO) and the lowest unoccupied molecular orbital (LUMO) is increased in different extents upon the interaction with fibril, resulting in the hypsochromic shift effect on the absorption for these chemosensors. For probes with the same polyene number n = 2, the absorption maximum calculated for probe 20 with a naphthalene ring as the electron donor is 595 nm, and that for probe 32 with a chalcone scaffold is 478 nm, showing a shift up to 117 nm in comparison with probe 20. This suggests that the donor moiety in a probe plays a dominant role on the excitation energy. Even though the theoretical results are quantitative discrepant when compared to experimental measurements, the increase in absorption wavelength with the spacer group length and the relative trend on excitation energy for this series of probes are reproduced satisfactorily. Note that effects of molecular aggregation and nuclear motion are not included in our treatment, which may contribute to the discrepancies.

To further reveal the character of electron transition involved in the absorption, the electronic dipole moment and the transition natures of the probes are collected in Table 1. According to the equation of *δ*, the absorption intensity is determined by the transition dipole moment and the excitation energy. The data on *μ_0f_* and *δ*_abs_ listed in Table 1 is coincident with the theoretical formula. The transition nature analysis on the excited states elucidate that the maximal absorption is relative to the transition between the frontier molecular orbitals. For most of the probes displayed in Table 1, the one-photon absorption maximum is contributed by the transition from HOMO to LUMO. Nevertheless, absorption maxima for probes 16 and 32 are related to the HOMO-1 to LUMO transition.

**Figure 6:**
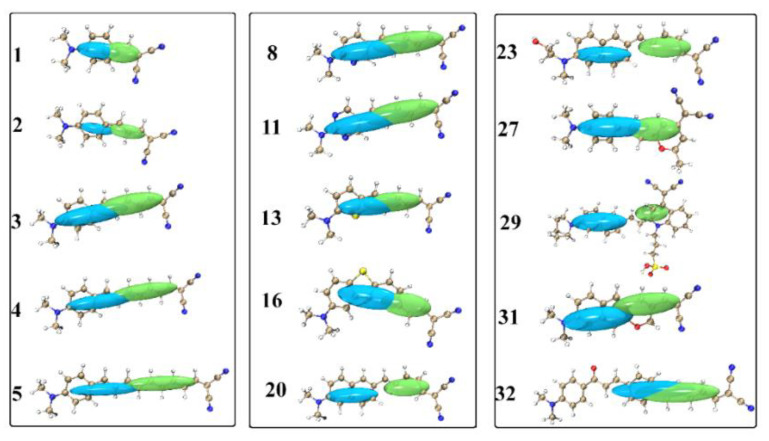
The hole (in blue) and electron (in green) distributions of selected probes caused by light absorption.

By utilizing the useful tool of hole–electron analysis, the electron excitation process for the probes is graphically shown in Figure 6. As can be seen, the absorption for the studied chemosensors involves a charge transfer from the electron-donating group to the electron-withdrawing group. However, the electron transfer distance and the degree of separation between the hole and the electron are obviously diverse for the probes. Particularly, in probes 20 and 29 with the naphthalene derivative as the electron donor, the hole–electron separation is much more evident than in other probes; and the hole–electron distribution of probe 23 with the similar electron donor part of probe 20 shows the same situation as in probe 20. On this basis, one can speculate that the absorption for the Aβ fluorescent probes is capable of being modulated by changing the donor moiety and the π-bridge connected to the dicyanomethylene segment.

### 2.3. β-Amyloid Induced Changes in Fluorescence

**Figure 7:**
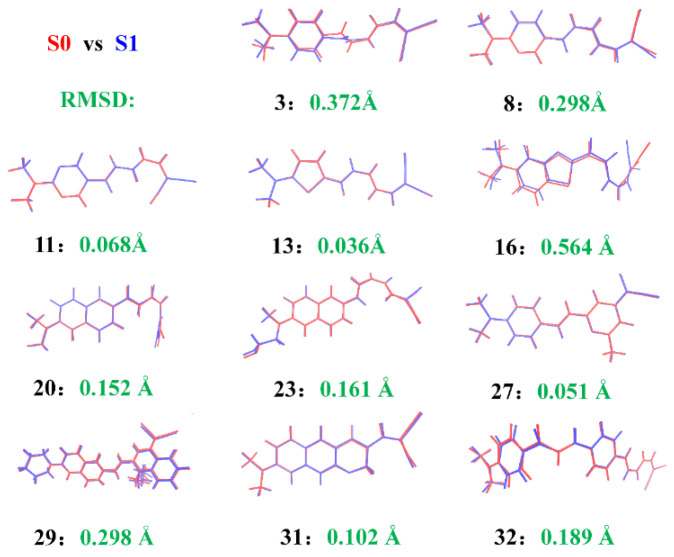
Geometrical comparison between the ground state (in blue) and the first excited state (in red) with RMSD values for selected probes in Aβ.

Concerning the application as fluorescent probes, it is desirable to have an intense fluorescent response for the probe when binding with Aβ. For this reason, the excited state dynamics are investigated by optimizing the first excited state for the probes in aqueous and in Aβ fibril. Upon excitation, the molecule will absorb the photon energy and one electron will be transferred, leading to differences in molecular geometry and electronic structure. Conformational changes between different states for partial probes are quantitatively described by RMSD as shown in Figure 7. Compared with other probes, the conformation of probe 16 undergoes the most obvious structure variation with a RMSD value of 0.564 Å. However, the geometry varies little for the probes 11 (RMSD = 0.068 Å), 13(RMSD = 0.036 Å), and 27(RMSD = 0.051 Å), which represents a rather stable configuration in different states in the fibril.

**Figure 8:**
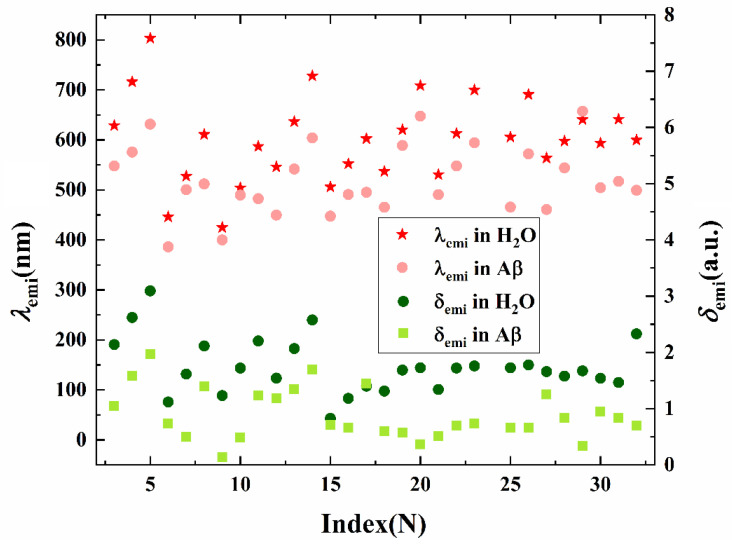
The fluorescent wavelength *λ*_emi_ (the left vertical axis) and emission strength *δ*_emi_ (the right vertical axis) for probes 1–32 in H_2_O and Aβ.

Since the conformation is extraordinarily sensitive toward the molecular optical properties, the photoemission properties of the studied probes in Aβ are subsequently explored and the results in H_2_O are also given in Figure 8 and Table 2 for the purpose of making a comparison. In the present case, a blue shift of the fluorescent wavelength in Aβ is seen for almost all probes compared with that in water, whereas in probe 29 a slight red shift from 640 nm to 657 nm is seen. Moreover, these series of compounds show an obvious variation on the fluorescent intensity when binding in β-amyloid except for probe 17, indicating their practicable usage as promising chemosensors applied in fluorescence microscopy for imaging Aβ.

The emission peak calculated for probes 3, 8, 11, and 13 are 629, 611, 581, and 636 nm in H_2_O, respectively, and the values shift to 548, 512, 483, and 542 nm in the fibril. This indicates the fibril environment induced changes and a donor effect on the emission properties of the probes, following the case of our previously reported results [27]. The situation also occurs for probes 16, 20, and 23, where the fluorescence maximum in H_2_O corresponds to 522, 709, and 700 nm, respectively, and blue shift to 491, 648, and 594 nm in Aβ. It is notable that the maximum emission wavelength for probe with a similar architecture but an extended polyene chain exhibits a bathochromic shift with enhanced absorption intensity in H_2_O. Due to the interaction with the fibril, the fluorescent peak shows a blue shift and the intensity is reduced compared with those in aqueous. Overall, the conformational and the microenvironment significantly contribute to the fluorescent property of the probes, which is consistent with the experimentally reported observations on the trend [9,19,23,24,25] in spite of some discrepancies on the value. The numerical difference may result from the flexibility of the amyloid, which we have not considered in the study. Although more accurate results will be obtained with the consideration of the dynamic evolution of the amyloid; here, we focus on the most stable probe–fibril conformation in order to make comparison among the probes at the same theoretical level and to further propose available structure–property relationships for these probes. Moreover, different convergences between the theoretical simulation and the experimental observation were found for different probes. For example, the calculated and experimental values for probe 11 in H_2_O are convergent, while probe 16 shows an evident discrepancy both in H_2_O and Aβ. The difference in convergence for different probes probably originates from the PCM model only covering a long-range interaction, while ignoring the vibrational contribution, the hydrogen bond with the solvent, and the complicated interaction between molecules and all binding sites, etc. Especially, the fluorescence enhancement was observed in the corresponding experimental works, whereas the calculated photoemission intensity in Aβ was smaller than that in water. Thus, restricted geometry changes in the fibril microenvironment can be determined and the favorable planarity of the probes is achieved in an aqueous solution. It also should be noted that only the most likely binding site with the largest binding affinity is taken into consideration in the calculation. In fact, various binding sites for a probe are available, and the average intensity over all sites may be experimentally measured.

In addition to the fluorescent wavelength and intensity, both the Stokes shift and the fluorescent lifetime are important parameters to characterize the applicability of a probe. Considering this point, a Stokes shift and a fluorescent lifetime calculated by the Einstein transition formula (in a.u.) *τ* = c^3^/(2*E*_emi_^2^*δ*_emi_) are listed in Table 2. Here c is the light velocity, *E*_emi_ and *δ*_emi_ denote the transition energy and oscillator strength, respectively. A large Stokes shift in the Aβ probes are demonstrated both in H_2_O and in the fibril, thus the overlap between absorption and emission spectra is small, avoiding the reduction of fluorescence efficiency caused by energy transfer. In addition, the emission lifetime for the probe in the fibril is located in the nanosecond time domain. Thus, a fast emission process can be expected, which results in facility of fluorescent detection for Aβ. Although the fluorescence peak is blue shifted in the fibril for the probes except for probe 29, the fluorescent oscillator strengths are decreased, while that of probe 29 is increased (see Figure 8). Therefore, compared with the case in Aβ, fluorescence lifetimes of all the listed probes but probe 27 are shorter in water than those in the fibril. Especially, the synergistic effect of emission energy and intensity results in a longer lifetime of probe 27. It should be noted that both the emission energy and the oscillator strength of molecular fluorescence can influence the fluorescence lifetime of the probe according to the above-mentioned equation.

**Figure 9:**
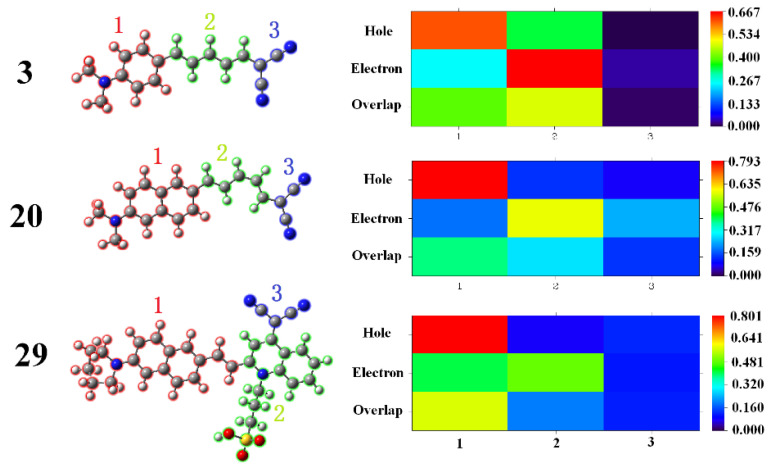
The partitions of probes 3, 20, and 29 and the electron–hole distribution heat maps for the molecular in the first excited state.

For the purpose of illustrating the transition natures in the emission process, the hole and the electron distributions for probes 3, 20, and 29 are calculated representatively based on the first excited state geometry. The corresponding heat maps are presented in Figure 9. The excited electrons for probe 3 mainly come from fragment 1 (labelled in red), and most of them are transferred to fragment 2 (labelled in green). The overlap of the hole and the electron is localized on both fragments 1 and 2. In the case of probe 20, the overlap of hole and electron is relatively scattered. The majority of the excited electrons comes from fragment 1 are transferred to fragment 2, and a small part are transferred to fragments 1 and 3 (labelled in blue). For probe 29, the delocalization of the hole is smaller than that of the electron. The electron is mainly distributed on fragment 1 and approximately one half is transferred to fragment 2, resulting in a relatively higher overlap degree at fragment 1 and thus remarkable oscillator strength. All of these results are in accordance with the above-mentioned results in Figure 8 and Table 2.

On the basis of the dynamical analysis on the excited state, we tried to establish the structure–property relationship for the probes. Firstly, extension of π-conjugation can largely affect the fluorescence wavelength. In general, a red shift is observed in emission maximum with the increase of spacer, which has been experimentally reported elsewhere [18]. For example, our calculations show that the fluorescent wavelength of probe 11 (polyene chain length of n = 2) in the aqueous phase shows a red shift of 70 nm compared to probe 10 with n = 1, while in the fibril environment a blue shift from 489 nm to 482 nm is demonstrated. This reveals the influence of the microenvironment on fluorescence, which should be considered for the accurate prediction of the optical property. Secondly, the donor effect does contribute to the emission peak position: For compounds with the same spacer of n = 2 but different aromatic ring as donor, probes 20 and 23 with a naphthalene derivative exhibit a longer respective fluorescent wavelength of 648 nm and 594 nm, in comparison with other probes. Moreover, the calculated emission wavelength for probe 11 with a pyrimidine derivative as the electron-donating group shows a significant blue shift to 483 nm. Last but not the least, probes 20 and 29 respectively show greater fluorescent variation on the intensity upon binding to Aβ compared to the other probes with n = 2. These can be attributed to the electron delocalization of these two probes in the excitation process, as shown in Figure 6. On account of probe 20 possessing both a longer fluorescent wavelength and a more evident fluorescent response when interacting with the Aβ fibril, we can suppose that a naphthalene derivative acts as a preferable chromophore. To summarize, the balance among the electron donating/accepting ability of the fluorophore, the length of the conjugate, and the binding affinity mentioned in the Section 2.1 need to be comprehensive optimized for designing this type of probe with high efficiency.

## 3. Methods and Materials

Geometry optimization and electronic structure calculation for the molecular probes were performed using the density functional theory (DFT) method with the B3LYP functional and 6–31+G(d,p) basis set using Gaussian 16 [28]. Frequency calculations were implemented to insure the stability of the conformations. To reproduce the aqueous environment distributed widely in vivo and the experimental pH conditions, polarizable continuum model (PCM) with solvent of H_2_O is used in the calculations. Considering the information of binding sites was not available, we carried out blind molecular docking for the probes with full-length β-amyloid (PDB code: 5OQV) [29] using AUTODOCK software by adopting the semiflexible approach [30]. The number of grids were chosen as 126 × 126 × 76 with a spacing of 0.465 Å. 100 docking modes with high binding affinity were generated for each probe, and the amyloid–ligand complexes with the lowest binding free energy for each binding mode were selected for further analysis.

For complexes with the highest binding affinity among all configurations, the QM/MM method with a two-layer ONIOM model was adopted to optimize the geometry and to calculate the optical properties [31]. In this method, the probe is regarded as the high layer and treated by the QM method with B3LYP/6–31+G(d,p) level. Meanwhile, the Aβ fibril is selected as the low layer and calculated by the MM method with a universal force field (UFF). An electronic embedding scheme was used to associate the QM and the MM components. The amyloid-specific one-photon absorption (OPA) and fluorescence for the probes were acquired by employing the time dependent-density functional theory (TD-DFT) and first excited state optimization calculations, respectively, using the same approach as mentioned above. All these calculations were performed in the Gaussian 16 package.

In order to illustrate the electron excitation characteristics, geometry changes between different states of the molecule as well as the hole-electron distributions are analyzed based on the optimized structures of the probes. Thereinto, the root mean squared displacement (RMSD) with the expression $RMSD=\sqrt{\sum_{i}\frac{{\left(x_{i}-x_{i}^{\prime }\right)}^{2}+{\left(y_{i}-y_{i}^{\prime }\right)}^{2}+{\left(z_{i}-z_{i}^{\prime }\right)}^{2}}{N}}$ is adopted to quantitatively characterize the geometric changes. Here, *x_i_*, *y_i_*, *z_i_* and $x_{i}^{\prime }$, $y_{i}^{\prime }$, $z_{i}^{\prime }$ are the position of atom *i* in different conformations and *N* is the total number of atoms in the molecule. For the first excited state, the heat maps of the hole and the electron distributions are plotted. All of these analyses were realized by Multiwfn [32,33].

## 4. Conclusions

In this work, an integrated approach including molecular docking simulation, quantum chemical calculation and QM/MM method is adopted to systematically study the fibril-induced optical properties of versatile dicyanomethylene-based fluorescent probes for β-amyloid in Alzheimer’s disease. The important binding sites for the probes in the Aβ fibril are identified and binding modes are characterized to make a comprehension on the interaction between the fibril and the molecule. The conformation of the ligand–fibril complexes with a higher binding affinity are selected for optical property investigations. On the basis of the QM/MM calculations, lengthening the spacer tends to significantly induce a red shift in the optical spectra, which reveals the importance of molecular size on the optical property of the probes. In addition, the absorption maximum for probes with the same polyene chain ranges from 595 nm to 478 nm, corresponding to the probe with naphthalene ring and with a chalcone scaffold as the electron donor, respectively. Similarly, the shifts of the fluorescent wavelength (Δ*λ*) in different microenvironment for the studied probes is 61~106 nm, indicating the contribution of donor moiety on molecular photophysical properties. These results are in good agreement with the experimental measurements. To better understand the transition mechanism, the hole and the electron distributions are calculated based on the excitation process, which shows the variation on charge transfer for the probes with different electron-donating groups or π-conjugate length. Based on the results, an inner structure–property relationship for the studied probes is revealed. It is proposed that the comprehensive consideration on binding affinity, extension of π-conjugate, and the electron donating/accepting ability of the fluorophore should be combined to improve the efficiency of the dicyanomethylene-containing Aβ fluorescent probe. Importantly, the calculations indicate that naphthalene derivative can act as a preferable electron-donating group in the studied probes. Our work validates the feasibility of the employed integrated approach on capturing the fibril-induced changes in structure and optical properties of Aβ molecular probes, and the findings can bring a necessary theoretical insight on designing novel molecular probes for detecting amyloid fibrils in Alzheimer’s disease.

## Figures and Tables

**Table 1:** Absorption maximum *λ*_abs_ (nm), transition dipole moment *μ_0f_* (Debye), oscillator strength *δ*_abs_(a.u.), and the transition nature of selected probes in H_2_O and in Aβ.

Probe	in H_2_O				in Aβ				*λ*_exp_ *
	*λ*_abs_	*μ_0f_*	*δ*_abs_	Transition Nature	*λ*_abs_	*μ_0f_*	*δ*_abs_	Transition Nature	
1	400	32.73	0.98	H→L 98%	364	27.41	0.90	H→L 98%	429
2	464	53.31	1.37	H→L 98%	449	36.00	0.96	H→L 98%	482
3	526	76.62	1.74	H→L 98%	507	57.98	1.37	H→L 98%	507
4	583	101.40	2.08	H→L 98%	559	76.63	1.64	H→L 98%	519.5
5	638	127.40	2.38	H→L 98%	607	95.21	1.87	H→L 98%	531.5
8	508	72.69	1.71	H→L 98%	491	57.77	1.41	H→L 98%	465.5
11	481	73.94	1.84	H→L 98%	461	49.75	1.29	H→L 98%	439
13	525	78.93	1.80	H→L 98%	514	59.55	1.38	H→L 98%	588
16	474	33.45	0.84	H-1→L 98%	450	24.83	0.66	H-1→L 98%	559
20	595	60.37	1.21	H→L 98%	564	27.96	0.59	H→L 98%	599
23	588	61.48	1.25	H→L 98%	544	38.19	0.84	H→L 98%	512
27	484	48.78	1.20	H→L 98%	441	43.20	1.17	H→L 98%	464
29	527	38.08	0.86	H→L 98%	530	26.08	0.59	H→L 98%	500
31	550	43.52	0.95	H→L 92%	495	29.31	0.71	H→L 82%	500
	509	14.09	0.33	H-1→L 91%	464	15.72	0.40	H-1→L 82%	
32	478	87.09	2.18	H-1→L 95%	489	64.20	1.57	H-1→L 98%	466

* Experimental absorption wavelength measured in aqueous solution. H and L denotes the HOMO and LUMO, respectively.

**Table 2:** Fluorescent wavelength *λ*_emi_ (nm), Stokes shift (nm), fluorescent lifetime *τ* (ns), and the transition nature of selected probes in H_2_O and in Aβ. Δλ (nm) represent the shift in fluorescent wavelength of these probes in Aβ compared to the case in H_2_O.

Probe	in H_2_O				in Aβ				Δ*λ*
	*λ*_emi_	Stokes Shift	*τ*	*λ*_exp_ *	*λ*_emi_	Stokes Shift	*τ*	*λ*_exp_ *	
3	629	103	2.77	660	548	41	4.33	659	81
8	611	104	2.65	627	512	21	2.83	642	99
11	587	106	2.35	588	483	22	2.84	580	104
13	636	111	2.94	675	542	28	3.26	690	94
16	552	78	3.87	727	491	41	5.48	714	61
20	709	114	4.38	783	648	84	17.0	678	61
23	700	112	4.17	798	594	50	7.25	678	106
27	564	80	2.87	617	461	20	2.55	570	103
29	640	113	3.68	650	657	127	19.6	630	17
31	641	91	4.22	679	517	22	4.77	675	124
32	600	122	2.32	--	499	10	5.33	595	101

* Experimental fluorescent wavelength measured in aqueous solution and in Aβ.

## Data Availability

Not applicable.
